# Scaling Up Research on Drug Abuse and Addiction Through Social Media Big Data

**DOI:** 10.2196/jmir.6426

**Published:** 2017-10-31

**Authors:** Sunny Jung Kim, Lisa A Marsch, Jeffrey T Hancock, Amarendra K Das

**Affiliations:** ^1^ Department of Biomedical Data Science Geisel School of Medicine at Dartmouth Dartmouth College Lebanon, NH United States; ^2^ Department of Psychiatry Dartmouth-Hitchcock Medical Center Lebanon, NH United States; ^3^ Department of Communication Stanford University Stanford, CA United States; ^4^ Healthcare Effectiveness Research IBM Cambridge, MA United States

**Keywords:** opioid epidemic, opioid crisis, opioid-related disorders, substance use, substance-related disorders, prescription drug misuse, addiction, Facebook, Twitter, Instagram, big data, ethics

## Abstract

**Background:**

Substance use–related communication for drug use promotion and its prevention is widely prevalent on social media. Social media big data involve naturally occurring communication phenomena that are observable through social media platforms, which can be used in computational or scalable solutions to generate data-driven inferences. Despite the promising potential to utilize social media big data to monitor and treat substance use problems, the characteristics, mechanisms, and outcomes of substance use–related communications on social media are largely unknown. Understanding these aspects can help researchers effectively leverage social media big data and platforms for observation and health communication outreach for people with substance use problems.

**Objective:**

The objective of this critical review was to determine how social media big data can be used to understand communication and behavioral patterns of problematic use of prescription drugs. We elaborate on theoretical applications, ethical challenges and methodological considerations when using social media big data for research on drug abuse and addiction. Based on a critical review process, we propose a typology with key initiatives to address the knowledge gap in the use of social media for research on prescription drug abuse and addiction.

**Methods:**

First, we provided a narrative summary of the literature on drug use–related communication on social media. We also examined ethical considerations in the research processes of (1) social media big data mining, (2) subgroup or follow-up investigation, and (3) dissemination of social media data-driven findings. To develop a critical review-based typology, we searched the PubMed database and the entire e-collection theme of “infodemiology and infoveillance” in the Journal of Medical Internet Research / JMIR Publications. Studies that met our inclusion criteria (eg, use of social media data concerning non-medical use of prescription drugs, data informatics-driven findings) were reviewed for knowledge synthesis. User characteristics, communication characteristics, mechanisms and predictors of such communications, and the psychological and behavioral outcomes of social media use for problematic drug use–related communications are the dimensions of our typology. In addition to ethical practices and considerations, we also reviewed the methodological and computational approaches used in each study to develop our typology.

**Results:**

We developed a typology to better understand non-medical, problematic use of prescription drugs through the lens of social media big data. Highly relevant studies that met our inclusion criteria were reviewed for knowledge synthesis. The characteristics of users who shared problematic substance use–related communications on social media were reported by general group terms, such as adolescents, Twitter users, and Instagram users. All reviewed studies examined the communication characteristics, such as linguistic properties, and social networks of problematic drug use–related communications on social media. The mechanisms and predictors of such social media communications were not directly examined or empirically identified in the reviewed studies. The psychological or behavioral consequence (eg, increased behavioral intention for mimicking risky health behaviors) of engaging with and being exposed to social media communications regarding problematic drug use was another area of research that has been understudied.

**Conclusions:**

We offer theoretical applications, ethical considerations, and empirical evidence within the scope of social media communication and prescription drug abuse and addiction. Our critical review suggests that social media big data can be a tremendous resource to understand, monitor and intervene on drug abuse and addiction problems.

## Introduction

User-generated content and user interactions related to drug use (eg, opioid misuse) are prevalent and are rapidly emerging forms of communication across social media platforms [1,2]. Social media big data on this topic offer an avenue for observing and understanding the temporal trends of problematic drug use and relevant risk factors in real time [3], as well as the ability to measure the collective human behavior of an extremely large population of interest [4]. Social media big data involve large, digitized data resources that contain naturally occurring communication phenomena observable through social media platforms and that can be used in computational or scalable solutions to generate data-driven inferences. Not only do some people communicate and share personal experiences, questions, and thoughts about substance use problems on social media, but also persons with addiction problems seek out social support from others with similar addiction problems through networks and communications available on social media [5]. Davey and colleagues argued that online communities and forums are well suited for people to communicate about problematic drug use activities because of their presumed anonymity and relative freedom from geographic constraints and perceived stigma [6]. Shutler and colleagues noted that social media, such as Twitter, can be an observatory platform that can reveal patterns of the current opioid epidemic, as users tweet about illicit, nonmedical use of prescription opioids in part due to users’ perceived protection of their real identity [7].

Although well-established resources in the United States such as the Drug Abuse Warning Network and the National Survey on Drug Use and Health offer critical information about substance abuse practices, these traditional platforms are known to lag in time in terms of data availability to the public for possible use in research [3]. On the other hand, online communications regarding drug use problems (eg, opioid use disorders) are surprisingly prevalent on social media [8]. These unsolicited communication datasets provide researchers a novel opportunity to unobtrusively assess and track various health risks, human factors, and emerging trends surrounding drug use [3,9]. Daniulaityte and colleagues stated that online technologies have become one of the leading-edge sources for detecting patterns and trends in illicit drug use [3]. Miller and Sonderlund suggested that online communication technologies can be an effective means for communicating with hard-to-reach populations that have illicit drug use problems [10].

Along with advances in information and communication technologies, seeking health information, disclosing personal health concerns, and exchanging social support are pervasive forms of human communications on social media [2,11-20]. It is critical to develop a conceptual framework to enhance our scientific understanding of these naturally occurring communications on social media related to drug use problems. The use of social media data-driven knowledge may help researchers better identify the utility of these technologies for public health research beyond the knowledge gained from domain experts. Furthermore, computational findings that emerge from self-disclosed social media data may ameliorate concerns about research validity in self-reported data, namely in terms of social desirability, response bias, and memory recall biases.

Due to the analogous and reflective nature of one’s social media world to one’s own real world, user-generated social media big data are increasingly being embraced and analyzed to observe and predict psychological states and collective human behavior [21,22]. For example, researchers found that social media communications and profiles correlate with real-world reflection of the self [23,24]. Communication patterns and interactions on social media also predict narcissistic personal traits [25], psychological functioning and well-being [26], and personality traits [27,28]. In recent years, studies have demonstrated the utility of social media big data in understanding public health problems, ranging from mental health conditions [29], population-level influenza monitoring [30,31], pronounced use of cannabis concentrates in the marijuana-legalized states in the United States [32], and the prescription stimulant Adderall [2] to perceived risks and sentiment around marijuana use [17].

With a growing line of empirical evidence demonstrating social media’s usefulness for observing and predicting health behaviors, social media data on drug use–related communications are being analyzed to address various research inquiries, including temporal trends of problematic substance use [33,34], market changes, social norms and cultural aspects of drug use [35], public perceptions, and relevant psychological factors (eg, sentiments [7]). Systematically assessed results of these social media communication data at scale for drug use problems can further inform key outreach methods, future intervention components, harm-reduction methods, and control and prevention strategies, which, in turn, can be delivered via vital social media channels for public health promotion. To maximize the utility of social media big data in addressing the urgent public health problems (eg, substance use problems, the opioid crisis) in the Untied States, we present a conceptual framework designed to guide investigation on problematic drug use–related communications that are observable via social media and to advance their potential impact for public health outreach efforts.

### A Multidimensional Framework to Analyze Social Media Communications for Problematic Use of Prescription Drugs

Our conceptualization classifies the communication on drug use problems that is evident on social media into 4 key dimensions: (1) user characteristics, (2) communication characteristics, (3) mechanisms and predictors of problematic substance use–related communications on social media, and (4) psychological and behavioral consequences of these social media communications at an individual and a societal level.

First, understanding user characteristics (eg, who are the users that share drug abusive, risky behaviors and addiction-related psychological states on social media?) permits in-depth subgroup or moderator analyses of the drug-related communications and related risks [36]. More specifically, understanding the demographic characteristics of these communications can advance targeted monitoring of drug abuse trends, as well as enhance the development of moderation modeling for a specific subgroup categorization of interest [37,38].

Second, identifying the communication features of target social media big data can offer insights into temporal, linguistic, and psychological patterns associated with self-disclosed social media communications about problematic use of prescription drugs [35]. For example, Paul and Dredze led a promising study to develop data crawling and surveillance systems to capture contextual factors associated with recreational drug uses through data mining of online communication data [35]. They modified and used a factorial latent Dirichlet allocation, a multidimensional text modeling approach, to incorporate prior knowledge about contextual factors such as drug type (eg, cocaine), delivery method (eg, smoking), and outcome aspects (eg, effects, health). Their approach has demonstrated successful application of data informatics to capture and discover an arbitrary number of contextual factors that are clinically important to understand new recreational drugs and trends.

As emphasized in a recent study incorporating machine learning techniques on Twitter feeds for a mental health diagnosis, analyzing linguistic properties of self-disclosed communication on social media regarding substance use problems provides a novel opportunity to identify communication themes and unmet needs among people with substance use problems [39]. Understanding communication characteristics of these unsolicited social media data will facilitate timely treatment initiation and health communication outreach strategies. Furthermore, the communication properties can be examined in conjunction with analyzing the user characteristics of those communications to identify interacting subgroups of users who share specific topics or valence (eg, anger, fear) regarding problematic substance use.

Third, investigating why and in which contexts people use social media to communicate about substance use–related problems reveals the mechanisms of various unsolicited behaviors (eg, self-disclosing personal stories about the nonmedical use of prescription drugs; using social media to receive social support during the addiction recovery process). Identifying this mechanism of communication behavior through analyzing social media big data, along with user interviews and self-reported surveys, can inform a strategic mediating construct for investigators when developing social media-based prevention or intervention programs (eg, [2]).

Fourth, understanding the psychological effects (eg, self-disclosure) and behavioral outcomes (eg, social influence) that such unsolicited uses of social media communications have on the self and others has received increased public attention [40]. However, this area of research has not been fully explored with a focus on the practical and clinical potential of social media technologies to promote health outcomes [41-43]. Social media big data analytics, in conjunction with mixed methodologies involving longitudinal follow-up and cross-sectional surveys or qualitative interviews, may help researchers identify these potential outcomes (eg, the effects of using recovery support groups on Facebook). Examining the outcomes of naturally occurring social media communications can offer intervention models that target critical moments to deliver a just-in-time intervention via social media at scale.

Integrating these 4 themes into a multidimensional framework enables systematic observation of factors and conditions explaining pervasive uses of social media for drug use–related communications. Development of this multiconceptual framework can also help researchers and clinicians explore the predictive and mechanistic values of social media-based communications in delivering state-of-the-art drug abuse recovery support and engagement systems. Furthermore, learning outcomes relevant to this multidimensional framework will offer data-driven strategies for leveraging social media data, features, and platforms for health promotion (eg, campaigns), as well as for understanding the nature of human communications concerning time-sensitive health issues.

In this work, we begin by reviewing the current use of social media for problematic drug use–related communications. Then, we highlight ethical challenges and methodological considerations when using social media big data for research on drug abuse and addiction. Lastly, based on these 4 conceptual dimensions and ethical considerations, we provide a narrative summary of the literature on social media-based drug communications and propose a typology with key initiatives aimed at addressing knowledge gaps in the use of social media for research on problematic and nonmedical use of prescription drugs.

### Prevalence of Drug Use–Related Communication on Social Media and Its Social Impact

#### Substance Use–Promoting Communication and Its Impact

User-generated content that promotes substance use (eg, positively commenting on pictures of illicit drugs [44]) is prevalent across market-leading social media platforms, such as Instagram and Twitter [45]. Such content can diffuse rapidly and widely through easily accessible network ties within and across media platforms [44,46]. Capurro and colleagues conducted a systematic review of 73 studies that used social networking sites to understand various public health issues, including sexual risks and mental health [47]. The review reported that 86% of the reviewed studies described user-generated content and served as passive observational investigations for surveillance on target health events among hard-to-reach populations. Their review also implied that researchers are increasingly leveraging social media platforms and data within the domain of various public health issues, thus directly benefitting from the prevalence of user-centered data that indicate risky health behaviors and psychological states. This systematic review, however, did not report research that focused on social media big data–based findings for prescription drug addiction.

Moreno and colleagues [48,49] found that 41% of young adult participants had pictures or messages referencing alcohol, tobacco, or other drug use in their publicly accessible social media profiles. When social media users are frequently and repeatedly exposed to or engage in such substance-promoting communications, they may become more accepting of or immune to these risky behaviors. As noted in media and social influence frameworks, drug-use promotional communications on social media that are shared across social network ties can influence the exposed users to normalize the frequency of these behaviors and, as a result, may change their attitudes toward or risk perceptions of these substances (eg, [50,51]). A national survey of US adolescents found that 40% of all teens in a nationally representative sample had seen pictures on social media depicting other teens getting drunk or using tobacco or illicit drugs [46]. According to the National Survey of American Attitudes on Substance Abuse *,* adolescents who reported seeing pictures of peers using substances on social media were more likely to use substances than were those who had never seen this peer-generated content on social media [46].

Social psychology and media communication theories explain this link between content exposure and an increased willingness to engage in the behavior being promoted. For example, cultivation theory [52,53] posits that frequent media exposure to risky behavior influences the belief that the mediated version of reality is real, leading to overestimation of the frequency and prevalence of those risky behaviors in the real world. This distorted perception of reality leads people to accept risky or detrimental behaviors portrayed in the media, such as substance use, as relatively normal [46,54]. Social learning theory [55] further buttresses the claim that observing risky behaviors via social media can influence people to mimic behaviors or adopt specific values and thoughts. Bandura’s social learning theory [56] posits that media communication can considerably promote changes in human beliefs and behaviors by “informing, enabling, motivating, and guiding” the audience (pg 76).

Social media communication platforms allow substance users to connect with a wide array of social networks and readily accessible substance use–related content. A news feed on a social media site can become a platform that constantly provides both personalized and socially infused content for social modeling and mimicry. These socially mediated learning processes on social media underscore the importance of considering the consequences (eg, mimicry) of routine exposure to content that is positively framed for and, indirectly or directly, promotes problematic drug use.

#### Substance Recovery Support and Prevention Communication and Its Clinical Implications

While content that promotes substance use is prevalent on social media, use of social media networks and online communities to explore recovery support for drug addiction problems is an increasingly popular form of communication [42]. Social media-based platforms (eg, Facebook Groups and Pages) are generated and led by self-motivated users with a goal of sharing and providing social support for users who have substance use problems or are in addiction recovery processes. Recently published work led by Rubya and Yarosh examined the characteristics of video-mediated, peer-led synchronous online support communities for addiction recovery [42]. Although the platform they examined in their work is not one of the social media platforms in the current market (eg, Facebook, Twitter), it did offer social media components, such as user interaction features, social networking functions, and engagement tools, that are important for peer-based online social support [57]. Through data crawling, self-selected online surveys, and in-depth interviews, Rubya and Yarosh examined the role of video-based online forums for people in addiction recovery and reported that these forums were viewed as critical in helping people with opioid use disorders overcome any perceived barriers (eg, access, anonymity) to seeking recovery support for drug addiction.

Compared with their application in the drug addiction context, the values of peer support and user engagement on social media platforms have been leveraged in health interventions for other health contexts, such as smoking cessation [58] and weight loss among vulnerable populations [59]. For example, Kim and colleagues [57] used various Facebook Group features for a 6-week smoking cessation and reduction intervention. Their findings support a successful rate of smoking reduction predicted by user engagement (eg, the number of comments posted) and the amount of social support received (eg, the number of “likes” participants received). These mechanisms were facilitated within their Facebook Group intervention that was designed to assist regular smokers to quit cigarette smoking. As discussed in 2 recent systematic reviews on social media-based studies within the public health interest, social media technologies and features have not been fully applied or used for long-term, scalable investigations that can generate successful social network-based health diffusion phenomena for health promotions [47,58]. We further argue that, despite the potential value of unique technology features in social media platforms, most of these platforms have not been systematically leveraged to offer evidence-based content or scientifically guided support for people in opioid addiction recovery processes. Accordingly, the public health and clinical benefits of leveraging social media platforms for substance prevention and recovery support remain underexplored.

A national report released by the US Centers for Disease Control and Prevention stated that almost 48,000 deaths in the United States in 2014 were induced by potent substance poisoning, surpassing the number of deaths caused by motor vehicle accidents [60]. The US National Institute on Drug Abuse reported that the total number of substance use–related deaths in the United States increased since 2001, with as much as a 6.2-fold increase in the number of overdose deaths from heroin from 2002 to 2015 [61]. The substance use epidemic in the United States has caused detrimental consequences on both individual and societal levels that call for urgent research on understanding this phenomenon in a scalable, systematic fashion.

The proliferation of substance use prevention and addiction recovery support communications among social media users provides empirical resources to systematically analyze naturalistic, real-time communications data and to gain time-sensitive insights into substance use risk factors, behavioral patterns, and prevention and recovery processes. Applying big data informatics—the process of examining large unstructured data to discover hidden patterns and useful information [62]—to substance use prevention and recovery support-related social media data will help us gain important and novel knowledge regarding the characteristics of substance use–linked users and their health behaviors [63-65]. Given the prevalence of social media use among people with drug use problems, in addition to the potential opportunities for learning about relevant risk behaviors and factors via user-solicited communication data on social media, we posit that this novel approach will be of importance to the development of evidence-based frameworks that promote addiction prevention and recovery support.

With these clinical implications and opportunities for understanding and enhancing a sensitive health problem, the social media data that contain various levels of user information (eg, age, gender) and users’ expectations and nuances embedded in social media contexts require cautionary attention to ethical boundaries and practices. In the following section, we discuss key ethical principles, challenges, and considerations applicable in this novel research setting when incorporating user-centered social media communication data for behavioral health research.

### Researchers’ Responsibilities and Big Data Ethics in Studying Social Media Users With Substance Use Problems

The use of social media big data for informatics can provide population-level inferences, such as pattern or trend recognition [66] and natural language processing [67], for drug use–related behaviors and psychological states. When using publicly available and personally relevant communication data to understand human health behaviors and risks, special attention should be given to research ethics, especially for data concerning sensitive topics like drug addiction. A balance between ethical principles and scientific discoveries gleaned from novel technology tools should be actively sought and put into practice prior to designing and performing data mining and informatics. Mittelstadt and Floridi [68] explored research ethics-related themes that emerged from meta-analyses of big data–based studies. From those themes, we synthesize relevant principles applicable to each stage of research using social media big data to understand the drug epidemic. Those stages are identified as follows: (1) determining the scope of search for data mining or retrieval, (2) conducting subgroup analysis or follow-up investigation, and (3) disseminating and presenting social media data-driven findings.

#### Social Media Big Data Mining and Retrieval

Throughout the process of mining and retrieving social media big data, it is important to balance an understanding of data privacy from the user’s perspective beyond the needs of researchers who are interested in the data to provide broader public health benefits. Taking a user’s perspective (eg, on beliefs and priorities from a user’s mental state) can be a useful practice for ethical considerations. Perspective taking is an other-centered social cognitive process that involves simulation of and making inference about the target counterpart’s mind and cognitive state [69]. Accurately understanding a user’s mind and expectations requires taking their perspective, perceiving the situation through the lens of the users who generated these social media communications [70]. In the context of social media data mining, it is necessary to take the perspectives of users who self-disclose personal content on social media, as users’ expectations of the privacy of their social media posts can differ from researchers’ own perceptions regarding the privacy of the posts. For example, one possible scenario might be that Facebook users post personal stories about their addiction problems on “closed” Facebook Groups, expecting that the self-disclosed content will be kept within the groups. Researchers may perceive the content as part of the aggregated piece of information that is technically available to others who join the groups. In this specific scenario, there is a perceptual discrepancy between social media users and researchers regarding how much access researchers should have to the social media data for data mining and analysis. Given this perceptual discrepancy, failure to communicate research purposes and data protection plans up front is likely to leave users feeling deceived, uninformed, or manipulated [71]. Prior to data mining and retrieval, we encourage investigators to understand ethical expectations and the notion of moral harm, which is assessed by the risk level versus the values of scientific discovery from the research activities, from the perspectives of users in a context-specific manner. One method of building mutual understanding between users and investigators might be contacting the groups’ administrators to gain insight on in-group norms and user expectations. Researchers might ask for an introduction to the group members or coordinate an announcement within the groups to inform members of the group that researchers may use anonymous data (without disclosing personally identifying user information) in an aggregated manner for research purposes (eg, [72]).

To protect users’ privacy and understand their expectations on the use of their potentially sensitive social media content, such as drug use–related communications, we also encourage researchers to construct guidelines for case-specific scenarios concerning potential ethical issues. For example, a research program at Harvard University published a guideline booklet that reviews ethical norms, case reports, and concrete guidance to help researchers who use social media sites for patient recruitment and contact [72]. The guideline booklet provides a series of dilemma-type scenarios that can occur between researchers, participants, and different stakeholders when social media sites are used for patient recruitment. The guideline booklet also provides multiple case analysis results for each scenario. In line with the guideline booklet, we argue that the same ethical and regulatory practices used in traditional recruitment settings (eg, respect for privacy) can be applied to social media contexts. However, different operational implications should be considered, such as prospectively understanding sensitive values of the social media communities and their users, and the impacts of social media-based research methods on public trust [73]. Given the increasing research attention focus on use of social media for observatory resources and for health communication outreach and delivery platforms, there is a need for developing a concrete guideline on ethical research practices for use of naturally occurring social media communication data and social media-based networks and users for observational purposes and health outreach studies.

As demonstrated in Harvard’s guideline for social media use for recruitment research, scenario-specific solutions for ethical challenges on social media-based observational studies can be implemented through “cognitive rehearsals” (or scenario planning) prospectively, rather than retrospectively. Cognitive rehearsal is a cognitive behavioral technique that allows individuals to develop an effective set of responses to a critical event. A cognitive rehearsal approach [74,75] is based on the assumption that a person’s reaction to a critical event can be learned and enhanced through reading instructions and building a knowledge base of responses specific to the event. Through cognitive rehearsal techniques, individuals can subsequently adapt learned skills from scripted responses when they face the previously rehearsed event. Cognitive rehearsal approaches have been used as an intervention component to improve responses to impulse control issues and lateral violence [74]. These cognitive rehearsal techniques can be applied during challenging events specific to ethical dilemmas concerning big data use. By “rehearsing” situations and events in advance and generating instructive guidelines, investigators can be better prepared for such situations if they arise, thus establishing a healthy academic culture and mutual trust between the researcher and the community of interest. This scenario-specific, empathetic approach to examine social media big data can be applied to various data mining and retrieval activities, including developing protocols for data anonymization, implementing confidentiality and data protection plans applicable to data mining, and mobilizing plans for strategic communications with social media users for the informed consent process.

#### Subgroup Analysis and Follow-Up Investigation

Researchers’ responsibilities extend to maintaining data confidentiality and protection, and ensuring the transparency of research activities. These activities include not only data mining and analysis, but also subgroup analysis or follow-up investigation (eg, distributing surveys among subgroups, or invitations for interventions). Investigators may need to conduct follow-up surveys or interventions to address questions raised by big data–driven findings. Electronic data are track-(back)-able and technological advances can allow deidentified social media data to be reidentified. Therefore, failures in confidentiality or lack of transparency during the follow-up processes can cause great concern among users if their data were aggregated from social media without their prior consent. The ethical implications and social consequences of contacting individuals who were attributes of the big data–driven findings should be recognized by researchers and policy makers in relation to data protection and human autonomy issues in big data research, even as the attributes of individuals were imperceptible to the aggregate level of big data–driven knowledge [68,76]. Before pursuing follow-up investigation, researchers should consider the rights of subjects and weigh context-specific risk levels in comparison with the value of scientific discovery from the research activities. Additional caution should be used with respect to not only the confidentiality and privacy of reidentified subjects and data security and protection, but also to the compliance between research activities conducted for follow-up purposes and the policies and terms of use of relevant websites [72].

#### Dissemination of Social Media Big Data–Driven Findings

Emerging technologies and big data have ethical implications beyond those identified in accomplishing research aims. Researchers are also responsible for ethically disseminating the findings extracted from social media big data [77]. Even if the anonymization of individual-level data is successful, findings that describe participants by specific geographical characteristics, socioeconomic status, health condition, risky behavior, or a combination of these characteristics can cause discrimination and stigmatization of those groups, which in turn can raise group-level harm and risk [78]. The consequences of ignoring group-level harm (eg, increased stigmatization of patients in addiction recovery as a result of big data–driven findings reported in an academic article) are nontrivial and can affect broad members of the identified groups or community [79], including those who have opted out of the study. Group-level effects of reidentifiable anonymized data require further research exploration in terms of their social implications for the groups’ users (eg, group-level stigma and group-level privacy) and potential unintended discrimination against subgroups with particular demographic characteristics or health-related problems [80].

Ethical principles, including the fundamental rights of “autonomy, protection, safety, maximization of benefits and minimization of harm, and respect for beneficence” ([81], pg 4), are not difficult concepts to understand. However, applying these principles to a sensitive social media big data context (eg, substance use) can raise ethical challenges. Ethics protocols and guidelines for social media big data–driven health research are evolving relatively slowly, compared with the pace of research outcomes using social media big data. These challenges require special sensitivity to the dynamics of social network-based communities and can only be addressed by carefully engaging in iterative ethical decision-making processes, both prospectively in designing studies and retrospectively by learning from ethical practices conducted in the relevant literature.

## Methods

We developed a typology based on a critical review process [82]. The strength of a critical review is not only that the review process evaluates the previous body of work, but also that it results in models of thought that can offer a new phase of conceptual framework development [82]. The typology is focused on delivering a set of relevant previous studies in terms of 4 conceptual attributes: (1) the characteristics of users who engage in substance use–related communications for promotional or preventive and control purposes; (2) the nature of substance use–related communications, such as their valence, expressed sentiment properties, and patterns of interactions; (3) the social and psychological predictors and mechanisms of those social media communication behaviors; and (4) the effects of problematic substance use–related social media communications on users. This knowledge development method is designed to elaborate on and identify the utility of social media as a means of communication delivery and observational platforms for users with substance use problems. Developing a multidimensional framework can further help theorize and synthesize underlying factors and conditions that influence social media use behaviors among online communities for problematic substance use–related reasons [43].

In our critical review, 1 researcher (SJK) independently searched for relevant literature within the PubMed database and the entire e-collection theme of “infodemiology and infoveillance” of JMIR Publications which includes the *Journal of Medical Internet Research* and articles in other JMIR journals indexed with this topic (http://www.jmir.org/themes/69). The search keywords were “prescription drug abuse,” “nonmedical drug use,” “social media,” “social network sites,” “big data,” “data mining,” “social media data,” “informatics,” “machine learning,” “Twitter,” “Instagram,” and “Facebook.” The same researcher (SJK) then evaluated the titles and abstracts of published studies based on their topical relevance (ie, problematic or nonmedical use of prescription drugs) and included studies that used social media communication data and computational analytic methods (eg, data mining, social network analysis, and supervised or unsupervised natural language processing).

Given the scope of the review—that is, focusing on how social media big data can be used to understand communication and behavioral patterns of nonmedical or problematic use of prescription drugs— in our typology, we did not include studies that reported data from nonsocial media platforms (eg, online forums), problematic use of other substances (eg, alcohol, cigarettes, and cannabis), or noncomputational analytic methods (eg, survey only) (eg, [1,3,5,6,35]). Note that demonstrating the systematic nature of the article search and conducting quality assessment are not components of critical review, and, thus, a Consolidated Standards of Reporting Trials (CONSORT) diagram is not required. After screening a large number of searched articles within the PubMed database and the entire e-collection theme of “infodemiology and infoveillance” within the *Journal of Medical Internet Research*, we reviewed findings from 8 highly relevant studies that met all the inclusion criteria. We report empirical evidence from the studies in relation to the 4 research inquiries, the methodological and computational approaches, and ethical practices and considerations discussed in each study (Multimedia Appendix 1 [2,4,7,33,36,83-85]).

## Results

Multimedia Appendix 1 lists studies that met eligibility criteria and were closely related to the topic of interest, along with the 4 conceptual dimensions, as well as methodological and ethical domains.

### User Characteristics

The characteristics of users who shared substance use–related communications on social media were reported by general group characteristics, such as college students, youth and adolescents, Twitter users, and Instagram users. Different types and levels of substance uses tended to be associated with different demographic characteristics, including sex, age, and socioeconomic status [38]. The demographic and social-psychological characteristics of users can be classification markers for certain types and patterns of substance use–related communications on social media (eg, polydrug use tweets with positive action verbs). Understanding social media communication data with specific user characteristics can inform the division of subgroups when targeting just-in-time interventions, addiction recovery support systems, or antidrug recovery campaigns through social media. However, relatively less research attention was given to analyzing or incorporating user characteristics along with the analyzed communication characteristics.

### Communication Characteristics

All the reviewed studies examined various communication characteristics, ranging from geographic and temporal trends associated with nonmedical use of prescription stimulants to sentimental (eg, positive vs negative connotation, emotions), contextual (abusive vs therapeutic), and thematic (eg, feeling high) aspects of social media communications regarding problematic use of prescription drugs (eg, [33]). In a prominent work, Hanson and colleagues analyzed alternative motives and potential side effects of drug intake for nonmedical purposes (eg, as a study aid) through tweets matching [33]. Hanson and colleagues also examined how social network factors explained the nonmedical use of prescription drugs and relevant risk behavior [2]. The empirical evidence in the reviewed articles demonstrated that aggregated, time-stamped social media big data can reveal linguistic characteristics, interaction activities (eg, posting text or image content portraying substance use), needs of and thoughts on drug intake, social relations, and risk behaviors concerning problematic prescription drug and polydrug use.

### Mechanisms and Predictors

There was limited work on directly examining moderating or mechanistic factors of drug abuse–related social media communications. The predictors and mechanisms of social media communications for nonmedical use of prescription drugs (eg, attitudes, risk perceptions) were not directly investigated in the studies we reviewed, and thus remain largely underexplored. Applying use and gratification theory of media can shed light on what motivates individuals to share or engage with such content on social media and what kinds of media use gratification (eg, entertainment, sharing problems for moral support) these media activities provide for people with substance use problems [86,87]. Understanding these mechanisms of social media use for drug-related communications (eg, drug use–related activities and seeking recovery support) can produce clinical insights into how these features can be used in a clinically meaningful manner to fulfill the realistic needs of people with substance use problems. For example, people with substance use problems disclose and share information, and interact with others for substance promotional and prevention- and recovery-related purposes. These self-initiated communication activities might be associated with psychological deficits or the need for social support among substance users, or might coincide with the finding that substance users find self-disclosure activities therapeutically rewarding [57,88]. Mechanistic investigation is likely to help researchers and clinicians identify key factors when considering the designs and development of an intervention to effectively treat substance use problems.

### Outcomes

The psychological and behavioral consequences (eg, increased behavioral intention for mimicking risky health behaviors) of engaging with and being exposed to social media communications regarding problematic drug use is another area of research that has been understudied. This dimension was not directly examined or empirically identified in the reviewed studies. However, the importance of understanding the varied aspects of these outcomes was discussed in some studies [7,33]. For example, Shutler and colleagues discussed the potential presumed effects of normalizing illicit drug use behavior on social media [7]. Hanson and colleagues discussed how the prevalence of tweets about nonmedical use of Adderall (eg, as a study aid) may produce a misperception that risky drug use behavior is acceptable among peer groups, and that, in turn, may lead to socially normalizing abusive drug behavior and increasing the levels of abuse [33].

Understanding short-term and long-term effects of media exposure and engagement for problematic drug use–related communications may require population-level-based surveys or longitudinal follow-up investigation in addition to social media big data analytics. By examining the psychological and behavioral effects of using social media for drug promotional or prevention- and control-related purposes, investigators can be well positioned to improve their ability to develop theoretical and methodological models when harnessing social media platforms for health promotion targeting public health problems, such as nonmedical and problematic use of prescription drugs. Investigating the outcome dimension will also help researchers understand the clinical implications and the utility of social media as behavioral intervention platforms.

### Methodological Domain

For the data mining process, some studies developed and tested their own social media data monitoring and crawling systems. For example, Cameron and colleagues developed the Prescription Drug Abuse Online Surveillance and Epidemiology (PREDOSE) infrastructure to extract and analyze entities and sentiments of unstructured social media text data regarding prescription drug abuse [83,89]. Data collection periods varies across studies, ranging from 2 weeks to more than 1 year (eg, [7,36]).

With the exception of 2 studies conducted by Correia and colleagues [4], who used Instagram data, and Cameron and colleagues [83], who did not disclose the name of the analyzed social media platform, all other reviewed studies analyzed Twitter data. Also, with the exception of the Hanson et al study [2] that explored social networks, most of the reviewed studies examined the linguistic properties of Twitter communication data. In doing so, they applied different machine learning (supervised or unsupervised learning) models to a random subset of filtered Twitter text data to identify common latent themes, patterns, and sentiments associated with nonmedical prescription drug or polydrug use (eg, [84]). A study led by Hanson and colleagues examined social networks of nonmedical or abusive use of prescription drugs and polydrug use among college students by selecting 25 subsets of tweet networks that comprised 2227 unique Twitter users. They explored social circles and interaction patterns within each network [2]. They used mixed methods involving human coding, qualitative content analysis, and manual annotation tasks, along with filtered keyword searches, as part of an iterative process to precisely understand a large volume of Twitter content promoting nonmedical use of prescription drugs, such as opioid analgesic drugs [7,33,36,84,85].

### Ethical Domain

Among the reviewed studies, 4 reported that an institutional review board (IRB) approved their study [2,33,83]. Two studies reported that the IRB review was waived or was not applicable [7,36]. The status of IRB review and approval was not explicitly reported or discussed in some studies [4,84,85]. The reviewed studies used either Twitter text data or Instagram data, and these datasets were considered publicly open sources, although the topic of interest was problematic use of prescription drugs, which can be personal and risk sensitive. Some researchers reported ethical practices they applied in compliance with their IRB guidelines. For example, Cameron and colleagues [83] did not disclose the name of the social media platform they analyzed, and Kalyanam and colleagues [84] discussed the data anonymization process (eg, removing user name and profile information before analysis). In most studies, potential ethical issues and practices were not discussed in detail. This might be, in part, because the social media data in their studies was considered publicly open or because discussing ethical aspects was not directly within the scope of their study.

## Discussion

The detrimental consequences of substance use highlight the urgent need for research to understand the drug epidemic in a scalable, systematic manner. Performing big data analytics on social media content allows researchers to generate data-informed insights into the phenomena of interest, such as the promotional communication of problematic substance use shared on social media platforms. The use of social media data to monitor and observe problematic use of prescription drugs, such as nonmedical use of analgesic opioid drugs, is as yet an unexplored area of biomedical research. In our reviewed studies reported in a typology, the communication properties were used to identify high-risk events [90], indicating the potential utility of social media data as a resource in scaling up surveillance systems for substance use problems. More specifically, social media communication data aggregated by drug use–related search keywords can indicate the level and stage of drug dependence, the actions of patients engaging in addiction recovery support groups, former users with or without relapse episodes, or current users with or without dependence. Given the large scale of social media communications posted by people who have engaged or are engaging in nonmedical use of prescription drugs such as opioids, harnessing social media platforms and data will provide insight into important novel discoveries of collective public health risk behavior.

In this paper, we propose a multilevel framework and ethical considerations that are applicable to social media communication data to understand problematic drug use phenomena. Based on the 4 dimensions in the framework, along with methodological and ethical domains, we conducted a critical, narrative review of empirical findings that were based on social media communication data involving problematic use of prescription drugs. The 4 primary conceptual dimensions are (1) understanding characteristics of users who share their data (eg, texts, pictures) about nonmedical substance use on social media; (2) the communication characteristics of such self-disclosed data; (3) predictors and underlying mechanisms of social media communications on problematic use of prescription drugs; and (4) the psychological and behavioral consequences that social media use for problematic drug use–related communications may have for users themselves (eg, active users) or others (eg, observers, lurkers). The state of social media uses among people with substance use problems is receiving increased attention. We designed an evidence-based, multiconceptual framework in our typology to inform potential future research directions, which may also offer insights into public health outreach strategies, as well as the development of social media-based substance use prevention and recovery intervention programs.

Advances in communication technology and informatics offer novel opportunities for understanding substance use problems through naturally occurring, self-disclosed communications on social media. This research requires multidisciplinary collaborative efforts between data scientists, social scientists, and clinicians to systematically structure and identify the ongoing substance abuse problems observable through problematic drug use–related communications on social media. Various social media features and characteristics (eg, easy access, perceived anonymity), automated analytic approaches at scale, and the prevalence of sharing and engaging user activities for such communications underscore the benefits of harnessing social media platforms and data to study drug use trends, patterns, and the underlying psychology and subsequent outcomes. Social media big data on the nationwide public health problem of nonmedical use of prescription drugs in the United States can also have a practical impact at the individual level (eg, seeking social support for addiction recovery support), as well as at the societal level (eg, public health campaign efforts on this topic). Although a different and extended set of ethical challenges exists in the realm of social media big data research, we expect that principle-guided, multidisciplinary, and iterative processes will soon start to converge for this topic. We also raise the importance of the precision and sensitivity of social media big data that can be prone to type I error (ie, falsely identifying and overgenerating inferences from data [3,91]). For future research, mixed methods incorporating survey research and recruitment strategies for longitudinal follow-up investigation can be used to improve the validity of social media data-driven findings.

We acknowledged a lack of theoretical frameworks that are applicable to social media big data for substance use monitoring and observational systems [91]. To bridge this gap in the research, we proposed a typology of the substance use epidemic that was traceable and observed through social media data. This knowledge synthesis is designed to analyze the state of the research on this topic and to guide future research directions. In this typology, we focused on previous work that used automated data analytics such as computational linguistic analysis and social network analysis, rather than using research outcomes purely based on manual coding of content analysis or noncomputational methods. The reviewed articles incorporated findings from supervised or unsupervised machine learning and various computational approaches. The reviewed findings demonstrated that surveillance systems incorporating social media data can produce comparable and valid findings in an epidemiological and scalable manner, in comparison with conventional survey and manual coding content analysis methods (eg, [33,84]).

In this paper, we aimed to offer key conceptual applications, ethical considerations, social media data-based empirical evidence, and a typology framework within the scope of dominant public health issues centered on nonmedical and problematic prescription drug use. As proposed in the novel typology, integrating the 4 conceptual dimensions and multidisciplinary research efforts may advance our knowledge on this nationwide crisis of prescription drug use problems in the United States. Future research may use the proposed conceptual framework and the perspectives delivered in this paper as a leverage in advancing scientific scholarship on this important topic.

## Appendix

Multimedia Appendix 1 A typology of social media big data analysis for prescription drug abuse and addiction research.

## Abbreviations

CONSORT: Consolidated Standards of Reporting Trials
IRB: institutional review board
PREDOSE: Prescription Drug Abuse Online Surveillance and Epidemiology
