# Supplementary material for: Scaling Up Research on Drug Abuse and Addiction Through Social Media Big Data
Source: J Med Internet Res. 2017 Oct 31;19(10):e353. doi: 10.2196/jmir.6426 (PMC5686417; doi:10.2196/jmir.6426)
Supplement: Multimedia Appendix 1 [file jmir_v19i10e353_app1.pdf]

A typology of social media big data analysis for prescription drug abuse and addiction research.

Dimensions	Conceptual Domain<br>User characteristics	Communication characteristics	Mechanisms and predictors	Outcomes and effects	Methodological Domain<br>Data analytic methods	Ethics Domain<br>Ethics reporting
Research questions	Who are the target participants studied?	What format, type, and theme of communications were analyzed in each study?	Why do people engage with social media for the target behavior studied?	What outcomes or effects were found as a result of social media-based communications for substance use problems?	What computational methods were applied, developed, and conducted for social media big data analysis?	What ethical practices were applied or discussed?
Hanson et al. (2013) [2]	Not reported, thus not applicable	Eight different types of risk and abusive behaviors regarding prescription drugs, including seeking, trading, and buying prescription drugs. Correlations based factors (eg, number of people in social circle that produced tweets on prescription drug abuse/risk behaviors)	Not directly studied, thus not applicable	Not directly studied	Based on Tweets mentioning prescription drug terms from November 29, 2011 through November 14, 2012; 25 Twitter user circles with prescription drug abuse terms were selected. Social circles of 100 people of these 25 index users were discovered. The tweets of the Twitter users in these networks were collected and analyzed.	IRB status reviewed and approved
Hanson et al. (2013) [33]	University and college student clusters	Thematic, geographic and temporal trends and nonmedical use or abusive patterns of prescription stimulant (Adderall). Co-ingestion with other substances and alternative motives of intake.	Not directly studied thus not applicable	User-reported side effects of Adderall abuse (sleep deprivation and suppressed appetite). Potential media exposure effects (eg, misperception and normalizing illicit behavior due to exposure to drug abuse-related tweets) were discussed.	Tweets with the keyword filter of "Adderall" were collected. GPS data were linked to a twitter user who posted content nearby college and university areas with a cutoff distance of 150 miles. The fully nested sequence of clustering was applied for grouping. Descriptive statistics were used to characterize the Adderall abuse, and ArcGIS 10 was used to visualize the intensity rates of GPS Adderall	IRB status reviewed and approved

					tweeters.	
Cameron et al. (2014) [83]	Social media forum users. Geographic and demographic information was not reported due to privacy restrictions of the social media forums	Texts are mined from 35,974 social media posts. Drug user knowledge, attitudes, and behaviors through the detection of temporal trends, entities, and sentiments	Not directly studied, thus not applicable	Not directly studied, thus not applicable	Developed and used PREDOSE for extracting and analyzing entities, relationships, and sentiments of unstructured social media text data on prescription drug abuse communications.	IRB status reviewed and approved. To comply with their IRB guidelines, the names of the social media forums are not disclosed.
Shutler et al. (2015) [7]	Not reported	Sentimental (eg, positive vs. negative connotation), contextual (abusive vs. therapeutic), and thematical (eg, feeling high) aspects of Tweets about prescription opioid use	Not directly studied, thus not applicable	Not directly studied, but discussed the presumed effects of "normalizing" illicit behavior on social media	Used Twitter Archiving Google Spreadsheet platform for data mining with prescription opioid-related keywords (eg, Oxycontin, Oxys, Vicodin). Analyzed 2,100 tweets collected from January 5 to 15, 2013 through an exploratory qualitative analysis with three coders.	IRB status - reviewed and waived.
Katsuki et al. (2015) [85]	Youth and adolescent Twitter users	Analyzed a large volume of Twitter content promoting non-medical use of prescription medications, and found 75.72% of the tweets with URLs were linked to an illicit online pharmacy that promoted the sale of Valium without a prescription.	Not directly studied thus not applicable	Behavioral or psychological outcomes of drug promoting tweets were not directly studied, thus not applicable	A total of 2,417,662 tweets were collected and analyzed through an iterative process of manual coding and supervised machine learning	Not discussed
Sarker et al. (2016) [36]	Not reported, thus not applicable	The prevalence, patterns and intents of abusing prescription medications (Adderall, oxycodone,	Not directly studied, thus not applicable	Not directly studied, thus not applicable	Data mining of tweets between March 2014 and June 2015 using the Twitter Streaming API; conducted qualitative and	Ethical approval and informed consent were reported as "not

		and quetiapine)			quantitative analysis to detect abuse and non-abuse-indicating tweets with a control medication (metformin) for comparison	applicable"
Correia et al. (2016) [4]	Instagram users who posted about prescription drugs for depression between October 2010 and June 2015	9,975 qualified posts among 6,927 users that mentioned FDA-approved drugs known to treat depression. Analyzed topics of the posts (eg, intake schedules, emotions, side effects)	Not directly studied, thus not applicable	Not directly studied, thus not applicable	Unsupervised network analysis of indirect and direct term associations using the proximity or the isomorphic distance graphs to understand drug-drug interactions, symptoms, adverse drug reactions, and associated natural products	Not discussed
Kalyanam et al. [84]	Not reported, thus not applicable	Discovered underlying latent themes regarding nonmedical analgesic drug use and relevant risk behavior (eg, polydrug abuse). Most of the themes described the use of more than one prescription drug, or use of other illicit drugs (eg, ecstasy, heroin), in addition to opioid drugs. Different polydrug combinations were mentioned with different emotion adjectives.	Not directly studied, thus not applicable	Not directly studied, thus not applicable	Collected 11 million tweets about commonly abused prescription opioid analgesic drugs (Percocet, OxyContin, and Oxycodone) posted between June and November 2015. A two-step process of identifying themes and filtering out noise tweets was performed three rounds. The Biterm Topic Model (BTM) was applied on the subset of tweets for each drug to detect themes and patterns in corpora of signal tweets.	IRB review process was not discussed. But any identify-able user informa-tion (eg, account names) was removed before data analysis
